# Non-Contact Detection of Apnea-like Breathing Cessations Using Laser Speckle Pattern Analysis

**DOI:** 10.3390/s26103042

**Published:** 2026-05-12

**Authors:** Ayuushi Dutta, Amir Shemer, Ariel Schwarz, Yossef Danan, Yevgeny Beiderman

**Affiliations:** 1 Faculty of Electrical and Electronics Engineering, Holon Institute of Technology, Holon 5810201, Israel; ayuushid@hit.ac.il; 2 Department of Electronic and Electrical Engineering, Jerusalem College of Engineering, Jerusalem 9515701, Israel; amirse@jce.ac.il (A.S.); arielsc@jce.ac.il (A.S.); yossefda@jce.ac.il (Y.D.)

**Keywords:** sleep apnea, respiratory monitor, speckle pattern, optical sensing, non-contact sensing

## Abstract

Sleep apnea is a prevalent sleep-related breathing disorder characterized by recurrent cessations or reductions in airflow during sleep. It significantly impacts the quality of life, yet current diagnostic methods like polysomnography (PSG) are expensive and uncomfortable, limiting accessibility and ease of use. We developed a novel non-contact biosensing system using secondary laser speckle pattern analysis and dedicated image processing algorithms for apnea-like breathing cessations. The proposed method was tested on 14 healthy subjects with diverse body characteristics, aged 22–50 years (mean 33.1 ± 9.3 years) and body mass index (BMI) ranging from 19.6 to 28.7 kg/m^2^ (mean 24.6 ± 3.0 kg/m^2^) at different ‘simulated’ sleeping positions (back-lying, stomach-lying and side-lying), using voluntary breath-holding protocols to simulate apnea-like cessations lasting 10–20 s (short duration) and 20–30 s (long duration). To evaluate the performance of the system without selection bias, two complementary five-fold cross-validation procedures were applied: a participant-level and a class-level stratification. Using class-wise stratification, the system achieved an overall accuracy of 87.0 ± 3.0% (95% CI: [85.3%, 88.7%]), long-cessation sensitivity of 91 ± 12.4% (95% CI:[83.8%,98.2%]) and a short-cessation sensitivity of 88.0 ± 11% (95% CI:[81.6%,94.4%]). The two-class classification strategy confirm the robustness of the approach, supporting the potential of secondary laser speckle pattern analysis as a low-cost, non-contact alternative for home-based sleep apnea screening.

## 1. Introduction

Sleep apnea is a major public health concern that affects millions around the world and is characterized by recurrent episodes of complete or partial obstruction of breathing during sleep. These breathing interruptions lead to oxygen desaturation, sleep fragmentation and a cascade of adverse health outcomes including sleep disorder breathing (SDB), cardiovascular disease, hypertension, stroke, diabetes and increased risk of accidents [1,2,3,4]. Obstructive sleep apnea (OSA) is by far the most common type of sleep apnea, approximately 84% of the cases, with nearly one billion people globally estimated to be affected by it [5,6]. This type happens due to the excessive relaxation of the pharyngeal muscles, leading to collapse of the surrounding soft tissues and either limit airflow (hypopnea) or completely obstruct breathing altogether (apnea), despite ongoing respiratory effort, leading to paradoxical chest and abdominal movements and loud snoring [7,8,9]. In contrast, central sleep apnea (CSA) represents a smaller but clinically significant subset, with a prevalence of approximately 1% among adults aged 40 and above [10]. CSA arises from impaired central respiratory drive in which the brainstem fails to initiate breathing, resulting in a complete absence of respiratory action, chest wall movement and diaphragmatic contraction [11,12,13]. Although CSA is associated with heart failure, stroke and neurological disorders, CSA is inherently less prevalent than OSA due to its distinct and less common pathophysiology; however, its reported prevalence is likely underestimated because of underdiagnosis and limited screening and diagnostic tools [10,11,14,15]. Mixed sleep apnea (also called as complex sleep apnea) features both OSA and CSA, affecting approximately 2.7% of the population [8]. The severity of sleep apnea is clinically quantified using the Apnea-Hypopnea index (AHI), which counts the number of apnea and hypopnea events per hour of sleep [16,17]. According to the American Academy of Sleep Medicine (AASM), sleep apnea is categorized as mild when the AHI < 14.9 events/h, moderate when AHI ~15–29.9 events/h and severe for AHI > 30 events/h [17]. It is important to mention here that an apnea event occurs when reduction in airflow ≥90% from the baseline and lasts at least 10 s. The 10 s duration threshold was established to exclude normal transient pauses in breathing and to ensure that the event is physiologically significant and long enough to induce measurable oxygen desaturation or sleep fragmentation [18,19].

Polysomnography (PSG) is the standard diagnosis tool for sleep apnea and remains the only definitive method to distinguish between OSA and CSA [20,21]. It involves simultaneous monitoring of multiple physiological parameters such as brain activity (EEG), muscle tension (EMG), heart activity (ECG), pulse oximetry for blood oxygen levels and respiratory parameters such as air flow measured via nasal cannula [22]. While this test is thorough, these laboratory-based PSG with multiple body-attached sensors are expensive, non-convenient, typically requiring hours of stay in specialized facilities with trained technicians and staff, limiting the accessibility for many patients [23,24,25]. Various other methods like portable monitoring systems and wearables have been developed as a way of countering the above limitations with PSG techniques [26,27,28]. Some devices like Continuous Positive Airway Pressure (CPAP) with integrated monitoring sensors can treat and monitor apnea events but these machines demand the use of masks, thus making it uncomfortable for the user [29,30,31]. Other wearable solutions based on photoplethysmography (PPG) offer convenience of use but exhibit high sensitivity to motion artifacts and provide only indirect measurements of respiratory events [32,33,34]. Another prominent example is EarlySense, which is a contact-free continuous monitoring system comprising a piezoelectric sensor placed under the patient’s mattress that detects mechanical vibrations transmitted through the mattress to continuously measure heart rate, respiratory rate, and body movement without any patient compliance or physical contact [35]. It has been validated against polysomnography, achieving 96.1% and 93.3% accuracy for heart rate and respiratory rate measurement respectively, demonstrating its clinical suitability for hospital and home-based sleep monitoring [35,36].

Recent breakthroughs in optical biosensing have opened new possibilities for non-contact physiological monitoring. Optical techniques are inherently non-invasive and can operate without physical contact with the subject, eliminating discomfort and reducing any form of tissue damage. Unlike electrical sensors, they avoid electrical safety concerns and are immune to electromagnetic interference, making them well suited for use in complex clinical environments as well as natural home settings. This makes them particularly suitable for continuous high-resolution monitoring and in vivo diagnostics across varied healthcare domains, underscoring the versatility and growing clinical utility of optical sensing approaches [37,38,39]. Among optical sensing techniques, laser-based methods have demonstrated great promise because of their high sensitivity to micro-motions and their capability to measure physiological signals remotely. Notable examples include laser Doppler vibrometry (LDV) which is based on the Doppler effect, where the frequency of the reflected light from a moving surface, like the chest, the abdomen, neck or upper airway, undergoes a shift proportional to the velocity of the motion [40,41,42]. However, LDV performance can be affected by the line-of-sight constraints, motion artifacts and system cost [43]. Another approach, called structured light or laser mesh, in contrast, projects optical patterns onto the body surface and analyze pattern deformations to extract respiratory motion over extended spatial regions, offering robust, multi-point measurement and enhanced tolerance to larger body movements, albeit with reduced sensitivity to very small vibrations and increased computational complexity [44,45,46].

Secondary laser speckle is a phenomenon in coherent optics, which occurs when spatially coherent laser light is reflected from an optically rough surface. The reflected light creates an interference or ‘speckle’ patterns on a camera due to the random phase distributions caused by the surface roughness [47,48]. Critically, these speckle patterns are highly sensitive even to nanometric movements of the illuminated surface and when applied to biological tissues, speckle patterns can detect subtle movements associated with cardiac pulsation, respiratory effort, and other physiological processes [49]. Laser speckle contrast imaging (LSCI) has been widely adopted in biomedical applications and is particularly successful in cerebral blood flow-imaging during neurosurgery and in assessing tissue perfusion in burn victims, where it measures blood flow by analyzing the temporal blurring of speckle patterns caused by moving red blood cells [50,51].

The proposed biosensing technique is based on the principle of defocused laser speckle pattern analysis [52]. In conventional focused imaging, motion of the illuminated surface produces rapidly changing speckle patterns in which lateral, axial and tilt components are inseparably coupled, resulting in complex speckles that are difficult to interpret. In contrast, deliberately defocusing the imaging system by operating in the far field such that the object is itself out of focus, causes the speckle pattern to exhibit a dominant lateral displacement rather than temporal intensity variations. Although the three types of object motion remain inseparable at object level, defocused imaging effectively suppresses the contributions of lateral and axial motion in the recorded speckle patterns such that the measurable lateral displacement of the speckle pattern is predominantly caused by the tilt motion. This far-field secondary speckle pattern analysis is thus a distinct application of speckle phenomena, where instead of analyzing speckle contrast changes over time (as in conventional blood flow imaging using LSCI), the spatial displacement of the entire speckle patterns is tracked to measure surface motion. This approach has been successfully applied to remote extraction of speech from vocal cord vibrations and cardiac signals from chest wall motion [52,53,54]. More recently, this technique has been extended to applications that demonstrate its versatility across diverse biomedical domains, including applications where speckle pattern analysis has been used for remote monitoring of visual cortex activity during shape recognition tasks [55] and for AI-powered speckle pattern detection of brain responses to clear versus incomprehensible speech [56].

The aim of this paper is to demonstrate the feasibility and accuracy of defocused laser speckle pattern analysis as a non-contact biosensing approach for the detection of physical presence or absence of chest wall movements in a diverse healthy cohort (with variations in age, body weight and body habitus), across ‘simulated’ sleeping positions (supine: back-lying; prone: stomach-lying; lateral: side-lying) using controlled voluntary breath-holding protocols. While the underlying physiological and neural mechanism that gives rise to OSA and CSA can be detected clinically only through techniques like PSG, our experiments serve to validate a novel non-invasive technique for identifying the mechanical signature of chest movements during apnea-like events.

## 2. Materials and Methods

### 2.1. Theoretical Explanation

The fundamental explanation in speckle-based vibration detection lies in the mathematical transformation that occurs when transitioning from focused to defocused imaging occurs [52]. In light propagation (Equation (1)), the speckle amplitude distribution $T_{m}$ at observation coordinates ($x_{0}$, $y_{0}$) is described by a Fresnel integral [57,58] where light propagating from the rough surface at distance $Z_{1}$ carries a quadratic phase term proportional to $\left[{\left(x-x_{0}\right)}^{2}+{\left(y-y_{0}\right)}^{2}\right]$, with x and y representing surface coordinates and λ denoting the optical wavelength. φ(x,y) represents the random surface phase distribution of the rough object.

(1) $$T_{m}\left(x_{0},y_{0}\right)=\iint \exp\left[i\varphi \left(x,y\right)\right]exp[\frac{\pi i}{\lambda Z_{1}}({\left(x-x_{0}\right)}^{2}+{\left(y-y_{0}\right)}^{2})]dxdy$$

This quadratic Equation (1) causes surface movements to produce chaotic, unpredictable changes in the speckle pattern, rendering motion tracking impossible. However, when the camera is defocused to observe at a far-field distance $Z_{2}$ (satisfying $Z_{2}>D^{2}/4\lambda$, where D is the illuminated spot diameter), and using axial approximation, the mathematical description transforms to Equation (2), where the phase term becomes linear, proportional to (${xx}_{0}$ + ${yy}_{0}$).

(2) $$T_{m}\left(x_{0},y_{0}\right)=\iint \exp\left[i\varphi \left(x,y\right)\right]exp[\frac{-2\pi i}{\lambda Z_{2}}(xx+{yy}_{0})]dxdy$$

Equation (2) is also called Fraunhofer approximation [57,58]. This linear phase relationship in space is described as surface tilts by angle α transforms by a two-dimensional Fourier transform to lateral shifts in the entire speckle pattern rather than random decorrelation. Now the system’s behavior is predictable, with the shift magnitude β being directly proportional to the tilt (β≈4πα/λ for small angles).

Since respiratory movements involve primarily tilt components (as the chest surface angles change during breathing), the speckle pattern displacement directly reflects respiratory motion. This respiratory induced motion was then quantified through frame-to-frame correlation analysis: for each consecutive frame pair in the spatial domain denoted as $I_{1}\left(x,y\right)$ and $I_{2}\left(x,y\right)$, two-dimensional Fourier transforms were computed, and cross-correlation was performed via the frequency domain.

(3) $$C\left(x_{c},y_{c}\right)=F^{-1}\{F\left\{I_{1}\left(x,y\right)\right\}F*\{I_{2}\left(x,y\right)\}$$

where F denotes a Fourier transform, and $F^{-1}$ is the inverse Fourier transform operators. The resulting spatial correlation map $C\left(x_{c},y_{c}\right)$ highlights the displacement between $I_{1}$ and $I_{2}$, by detecting the correlation peak. Sub-pixel localization of the peak was achieved via parabolic interpolation, enabling displacement measurement with sub-pixel precision. The sequence of peak positions across frames was compiled to generate trajectories in the x and y direction, represent the incremental motion of the speckle pattern due to chest surface movement.

### 2.2. Algorithm for Signal Processing

**Figure 1:**
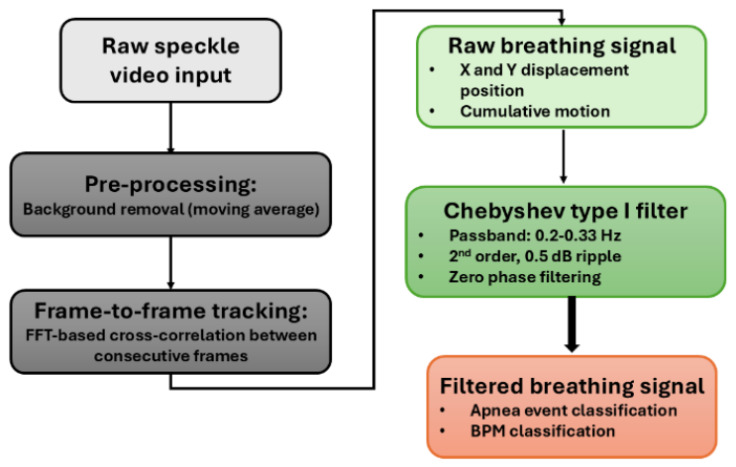
Signal processing pipeline for respiratory motion extraction and apnea detection using laser speckle pattern analysis.

Figure 1 illustrates the signal processing pipeline, where the recorded speckle videos were analyzed using a multistage MATLAB (R2024b) algorithm to extract the respiratory motion and identify apnea-like pauses in between normal breathing. Initially, a background reference was established from the first set of frames of the recorded video and background subtraction was applied to suppress static components while enhancing dynamic speckle variations associated with respiration (moving average). The sequence of frame-to-frame displacements was stored as a 2 × N matrix, where N represents the number of frames, with the first row containing horizontal (x-direction) displacements and the second row containing vertical (y-direction) displacements. Both axes were tracked simultaneously to capture the full two-dimensional chest wall motion during respiration. To obtain the cumulative position, the displacement vectors were integrated using a cumulative sum operation applied independently to each axis. The resulting signals contained both high-frequency noise and low-frequency drift components. To isolate the breathing frequency band, the raw position signal was processed using a second-order Chebyshev Type I bandpass filter with passband frequencies of 0.2–0.33 Hz (corresponding to 12–20 breaths per minute), representing typical human respiratory rates during sleep [59,60,61]. This step is carried out only for the horizontal component (x-direction) of the raw signal as it captures the predominant lateral chest expansion during respiration, typically exhibiting stronger signal-to-noise characteristics than the vertical displacement (y-direction). This comparison is explained in detail in Section 3.

**Figure 2:**
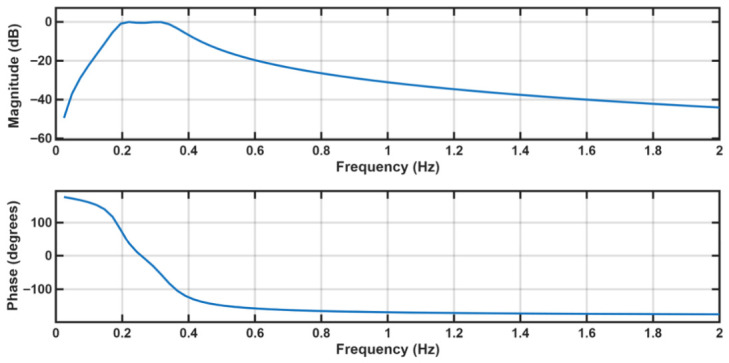
Frequency response characteristics of the implemented second-order Chebyshev Type I bandpass filter, demonstrating a sharp transition band and minimal passband ripple (±0.5 dB) within the target respiratory frequency range (0.2–0.33 Hz). The grid lines are for visualization.

Figure 2 shows the frequency response of Chebyshev Type I bandpass filter used in our signal processing protocol. The filter was designed with 0.5 dB passband ripple at a filter order of 2. The magnitude response of the filter confirms effective attenuation of frequencies outside the passband, with stopband rejection around −40 dB, ensuring that low-frequency baseline drift and high-frequency noise are adequately suppressed to preserve breathing signal components. This Chebyshev filter inherently introduces nonlinear phase distortion, particularly in. the transition bands, which could distort the temporal characteristics of the breathing events. To minimize these edge effects, the signal was mirror-padded with 20 samples at each boundary before filtering and zero-phase filtering was employed to prevent temporal distortion. This approach preserves both the sharp frequency selectivity of the Chebyshev design and the temporal integrity of respiratory waveforms necessary for accurate peak detection and apnea event timing. Individual breaths were identified using peak detection at a minimum inter-peak interval of 2 s (400 samples at 200 Hz). Pauses in between the normal-breathing signal were classified by multiple observers’ manual calculation over the reference BIOPAC device (explained in Section 2.3). Inter-breath intervals were automatically computed based on the duration of respiratory cessation. The overall respiratory rate was then computed as breaths per minute (BPM) by dividing the mean inter-breath interval across all detected peaks by 60. Visual outputs included time-series plots of the processed respiratory signal with annotated breath peaks and apnea-like cessation events, along with summary analyses displaying the respiratory, all of which is discussed in Section 3.

### 2.3. Experimental Design and Protocol

**Figure 3:**
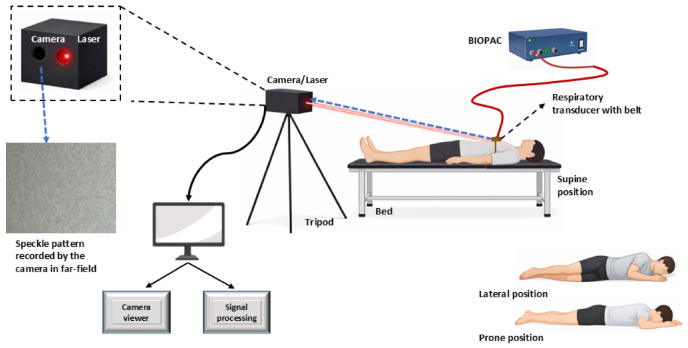
Schematic representation of the experimental setup for defocused laser speckle pattern–based sleep apnea monitoring of a volunteer sleeping in supine, lateral, and prone positions. A BIOPAC respiratory belt and acquisition system provides a reference signal for comparison.

Figure 3 illustrates the experimental setup of our non-contact respiratory monitoring system, along with simultaneous reference acquisition. The optical system comprises a continuous 5 mW, 780 nm wavelength, 30° divergence angle extended-source laser illumination, and a Basler high-speed camera (model: acA800-510 μm, by Basler AG, Ahrensburg Germany). operating at 200 frames per second, 256 × 128 pixels ROI (region of interest). The camera is equipped with a 75 mm focal length, 1-inch aperture doublet lens. Eye-safety for the extended source was calculated in accordance with IEC 60825-1:2014 [62], and compliance was approved by a certified laser safety officer. The entire system is enclosed within a single box and mounted on a rigid, height-adjustable tripod to ensure mechanical stability and that the recorded speckle pattern by the camera reflects the true motion rather than external noise or disturbance. The resulting speckle size on the camera was approx. 1 pixel. The laser illuminates the volunteer’s thoracic region at an incidence angle of approximately 65° and 70 cm from the laser, providing sufficiently wide and stable illumination, while maintaining subject’s comfort and eye safety. The camera is focused on the far-field regime to ensure speckle pattern displacement rather than total deformation and hence records the evolving speckle patterns with sufficient temporal resolution to capture rapid respiratory and chest movements.

Video acquisition is performed using Pylon Viewer software (v25.10), enabling control over key camera parameters such as frame rate, exposure time, and gain, while real-time histogram monitoring ensured proper exposure and prevented saturation of the speckle patterns during data collection. The recorded videos were subsequently processed using a custom MATLAB (R2024b) algorithm based on cross-correlation analysis to extract respiratory motion from the temporal evolution of the speckle patterns (as explained in Section 2.2).

For engineering validation and comparison required for chest-motion tracking, a reference respiratory signal is simultaneously acquired using a commercial respiratory transducer belt (SS5LB Respiration Belt) connected to an MP36 data acquisition system (BIOPAC Systems Inc., Goleta, CA, USA). The transducer is positioned around the chest of the volunteers as it measures the chest circumference during inhalation and exhalation, and the resulting temporal voltage signal serves as the contact-based reference signal for evaluating the performance of our experiments.

All recordings were conducted on healthy volunteers and were screened to exclude any known sleep-related or respiratory disorders that could confound baseline respiratory measurements. The participants were asked to lie on a bed and adopt common sleeping postures (supine, lateral, or prone). The cohort included 13 males (92.8%) and 1 female (7.1%) ranging in age from 22 to 50 years (mean 33.1 ± 9.3 years), with body mass index (BMI) from 19.6 to 28.7 kg/m^2^ (mean 24.6 ± 3.0 kg/m^2^).

Each volunteer completed three standardized sessions during a single experiment. In the first session, normal breathing was recorded for 60 s while the subject rested and breathed naturally without imposed control. This session served to establish baseline respiratory patterns and to verify the system’s ability to reliably track physiological breathing cycles. In the second session, controlled short-duration breathing cessation was induced over the 60 s recording period, where subjects initially breathed normally and then were instructed to voluntarily suspend breathing for 10–20 s following a verbal command from the operators, after which normal breathing was resumed. The third session followed an analogous procedure to induce controlled long-duration breathing cessation, with voluntary breath-holding durations of 20–30 s within a 60 s recording interval. To avoid confusion with frequency-based AHI severity categories of clinically diagnosed sleep apnea events, we classify our experiments according to breathing cessation duration as short duration (10–20 s) and long-duration (20–30 s). This duration-based classification evaluates our system’s ability to detect individual apnea-like induced cessation events in between normal breathing. Throughout all sessions, the BIOPAC reference signal was continuously monitored, and three independent observers verified the onset and termination of these artificially induced apnea-like events.

Ten participants were recorded in the supine position, while subsets of six and seven participants additionally underwent measurements in lateral and prone positions respectively, to evaluate robustness across common sleep postures. All experiments were conducted in a controlled laboratory environment under dim ambient illumination to approximate sleep conditions while maintaining optimal camera performance, with room temperature maintained between 20 and 24 °C. It should be noted that these conditions do not constitute actual sleep study. All the participants remained awake and followed verbal instructions from the operator during the experiments.

### 2.4. Performance Evaluation Protocol

To establish ground-truth labels for induced breathing cessations, three independent observers reviewed the simultaneously recorded BIOPAC signal and reached a consensus on the onset and offset of each breath-hold interval. According to the experimental protocol defined in Section 2.3., a breathing cessation is any continuous period of absent or near-absent respiratory effort lasting at least 10 s, during which the subject was asked to voluntarily hold their breath. This minimum 10 s rule aligns with both the experimental protocol and standard clinical definitions. The laser speckle signal was then compared against these reference intervals on a sample-by-sample basis at an acquisition rate of 200 Hz. For each processed speckle recording, individual breath peaks were first identified in the filtered x-direction signal using a peak detection algorithm with a minimum inter-peak interval of 2 s (400 samples at 200 Hz), as described in Section 2.2. The maximum breathing amplitude $A_{max}$ was then defined as the largest peak amplitude observed within the entire recording. The detection algorithm then identifies all continuous apnea-like intervals during which the speckle displacement signal remains below a normalized threshold fraction of $A_{max}$.

**Figure 4:**
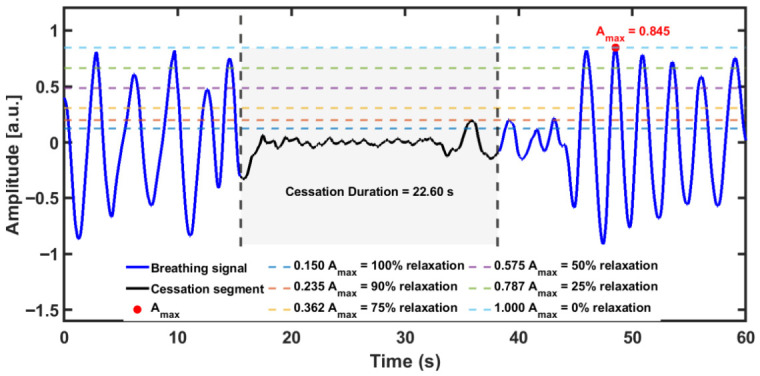
Threshold concept illustration using breathing signal from Subject S05. The figure overlays six threshold values on the same signal, illustrating how different threshold levels capture different fractions of the cessation region (shaded region).

Figure 4 illustrates the concept of the ideal normalized threshold using a breathing signal from Subject S05 recorded during a long-cessation session. The signal oscillates periodically during normal breathing, reflecting cyclic chest-wall motion, before flattening during a voluntary breath-hold and then recovering. The shaded region shows the interval that exceeds the 10 s minimum duration criterion. A normalized detection threshold is then set as a fixed fraction of $A_{max}$. Figure 4 overlays six threshold values THR = {0.15, 0.235, 0.3625, 0.575, 0.7875, 1.0} $\times \;A_{max}$ on the same signal, illustrating how different threshold levels capture different fractions of the cessation region. The figure clearly illustrates the practical consequences of threshold selection: a tight threshold (THR = 0.15 $\times \;A_{max}$) risks missing portions of genuine cessations at the onset and offset boundaries, reducing sensitivity and an overly permissive threshold (THR = 0.575 $\times \;A_{max}$) causes normal-breathing troughs to dip below the threshold for 10 s or more, generating spurious cessation detections and reducing specificity.

To identify the optimal detection threshold THR and evaluate classification performance without selection bias, two complementary five-fold cross-validation procedures were applied: a participant-level procedure in which all recordings from 20% held-out participants formed each test set and the remaining 80% as the training set, and a class-wise stratified procedure in which individual recordings were distributed across folds to maintain equal representation of normal breathing and short and long cessations in each fold (also in 20% test, 80% training ratio). In both procedures, threshold selection was performed exclusively on the training set of each fold, and the selected threshold was then applied to the held-out test set to obtain unbiased performance estimates. The threshold selection curves, per-fold performance metrics, and cross-method comparison are presented in Section 3.

The algorithm then assigns a single class based on the detected events. If the signal remains below the selected THR for at least 10 s, a cessation is identified, and its duration determines whether it is classified as short (10–20 s) or long (20–30 s). If no such interval is detected, the recording is labeled as normal. Shorter excursions (less than 10 s) are dismissed as normal breathing fluctuations. The four classification outcomes are defined as follows. A true positive (TP) occurs when a recording containing a cessation (either short or long) is correctly identified by the algorithm, with the detected event also matching the correct duration category. A false negative (FN) occurs when a cessation recording is missed entirely, meaning the signal does not remain below the threshold for at least 10 s and the recording is incorrectly labeled as normal. A true negative (TN) occurs when a normal-breathing recording is correctly classified as having no cessation event. A false positive (FP) occurs when a normal breathing is incorrectly flagged as a cessation, due to the signal dropping below the threshold for 10 s or more despite the absence of an actual breath-hold. Sensitivity represents the proportion of cessation events that are successfully detected by the algorithm and is calculated separately for short and long-cessation recordings.

(4) Sensitivity=TP/(TP+FN)×100%

On the other hand, specificity (temporal true negative rate) reflects the proportion of normal-breathing cases correctly identified as having no cessations and is computed only normal breathing.

(5) Specificity=TN/(TN+FP)×100%

Sensitivity and specificity exhibit opposing dependence on the threshold: as the threshold relaxes toward $A_{max}$, more of the cessation boundary is captured (increasing sensitivity) but normal-breathing troughs may also trigger detections (reducing specificity). The overall accuracy therefore peaks at an intermediate threshold that balances these competing demands.

(6) $$Accuracy=\frac{\left(TP+TN\right)}{total\;number\;of\;recordings}\times 100\%$$

In the context of respiratory event detection, false negatives (missed apnea events) are more critical than false positives, as they can lead to underestimation and inaccurate characterization of breathing interruptions. Thus, the threshold selection algorithm prioritizes the detection of all relevant events, even at the cost of a moderate increase in false positives by choosing an optimized threshold that would maintaining high sensitivity while preserving acceptable specificity rate. To provide a single summary metric that accounts for performance across all three classes (short cessation, long cessation, and normal breathing) without bias toward the majority class, the Macro-averaged F1 score was computed. For each class c, the F1 score is defined as the harmonic mean of precision and recall, expressed as

(7) $${F1}_{c}=\frac{2{TP}_{c}}{2{TP}_{c}+{FP}_{c}+{FN}_{c}}$$

The Macro F1 score is then computed as the unweighted arithmetic mean of the per-class F1 scores across all classes:

(8) $$Macro\;F1=\frac{1}{3}\sum_{c=1}^{3}{F1}_{c}$$

where c=1, 2, 3 indexes the short-cessation, long-cessation, and normal-breathing classes respectively, and the total number of classes is equal to 3.

## 3. Results

**Figure 5:**
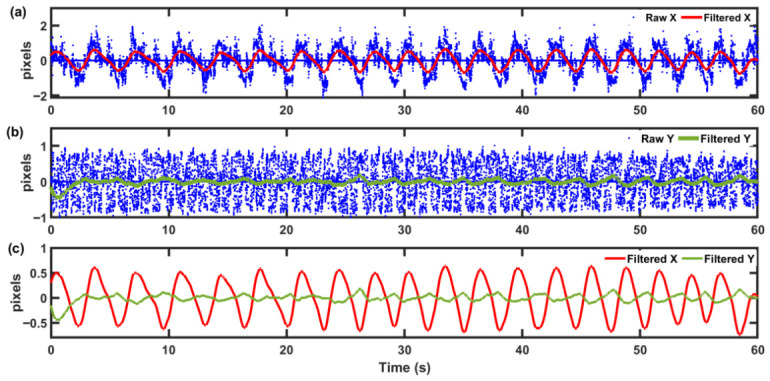
Temporal comparison of raw and filtered respiratory signals. Raw (blue dots) and Chebyshev Type I bandpass filtered signal in the (**a**) x-direction (red line) and (**b**) y-direction (green line). (**c**) Comparison of the filtered signals in the x and y directions, highlighting the stronger and more consistent respiratory oscillations in the x-direction.

As explained in Section 2.2, a second-order Chebyshev Type I bandpass filter was designed with passband of 0.2–0.33 Hz, corresponding to respiratory rates of 12–20 breaths per minute, which encompasses the typical range observed during sleep. The effectiveness of the bandpass filtering is illustrated in Figure 5 through a temporal comparison of the raw and filtered respiratory signals in both x and y directions. The blue dotted points represent the original raw position signal without any filtering, exhibiting substantial baseline drift, high-frequency noise, and motion artifacts superimposed on the periodic breathing pattern. In contrast, the solid curves show the final filtered signal after the complete signal processing pipeline (explained in Section 2.2) demonstrating successful isolation of the respiratory component with clear sinusoidal-like oscillations corresponding to individual breath cycles. The filtering effectively suppresses low-frequency drift and high-frequency noise while preserving the amplitude and temporal characteristics of the respiratory waveform. This preservation is essential for reliable peak detection in breathing cycles. The relative motion amplitude between the horizontal and vertical directions was quantified using the RMS ratio of the filtered signals, yielding an x/y ratio of 4.91, indicating a strong dominance of motion along the x-axis. This ratio holds similar values for all the subject recordings. Therefore, only the filtered x-direction signal is used for subsequent breathing signal processing and breathing-cessation analysis across all volunteers.

**Figure 6:**
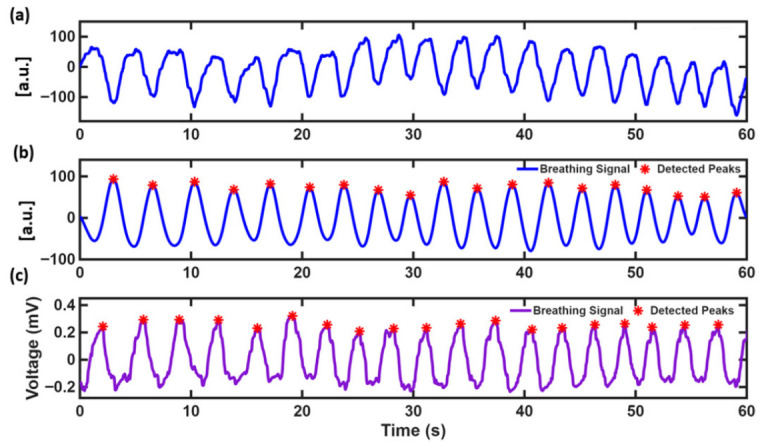
Respiratory signal processing stages and validation against BIOPAC reference (Subject S01). (**a**) Derived cumulative chest position signal, (**b**) Chebyshev-filtered signal (19.15 BPM detected) applied on (**a**), showing isolated respiratory oscillations and (**c**) BIOPAC reference signal at a sampling rate of 10 msec/sample demonstrating excellent agreement with the proposed system.

To assess the physiological validity and accuracy of the proposed sensing approach, the respiratory signals acquired using the laser speckle-based system were directly compared against simultaneously recorded reference data from a BIOPAC respiratory monitoring system. Figure 6 shows the data from Subject S01 during normal-breathing conditions over a 60 s recording period. Figure 6a shows the cumulative chest position signal, derived from the integrated frame-to-frame speckle raw displacements position. This signal captures both the respiratory oscillations as well as the underlying low-frequency drift from gradual chest movement over the recording period. Figure 6b illustrates the final processed breathing signal after Chebyshev Type I bandpass filtering (0.2–0.33 Hz) applied from Figure 6a, with detected respiratory peaks marked by red asterisks. The filtering success fully eliminates baseline drift and isolates only the respiratory frequency band, resulting in a cleaner sinusoidal waveform with consistent amplitude and well-defined peaks suitable for automated breath detection. Figure 6c shows the corresponding reference respiratory signal simultaneously recorded from the BIOPAC system at a sampling rate of 10 msec/sample. The laser speckle-derived breathing signal shows well-defined periodic oscillations, with closely matching phase and temporal features. Positive and negative deflections correspond to inhalation and exhalation, respectively. The algorithm calculated a breathing rate of 19.15 BPM from the laser speckle signal, in close agreement with the BIOPAC reference measurement (19 BPM), demonstrating the quantitative accuracy of the proposed system. No breathing-cessation events were detected during this recording, confirming accurate identification of normal respiratory activity without false-positive detections.

**Figure 7:**
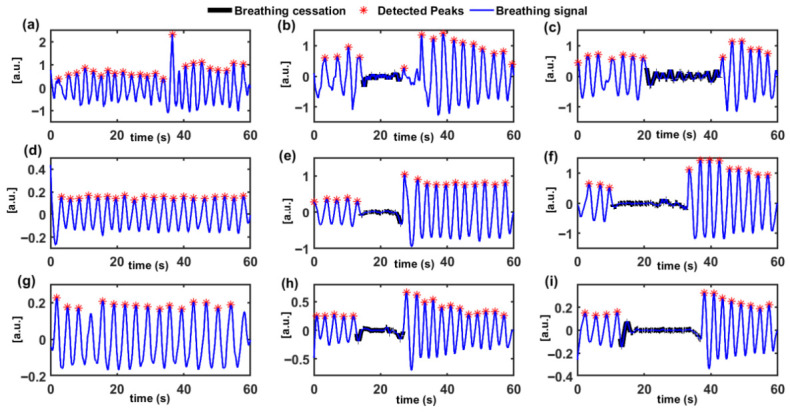
Position-dependent respiratory signal characteristics during normal-breathing and apnea events of Subject S03. (**a**–**c**) Supine: normal, short-duration, and long-duration apnea. (**d**–**f**) Lateral: normal, short-duration, and long-duration apnea. (**g**–**i**) Prone: normal, short-duration, and long-duration apnea-like events. Blue curves depict normal breathing, with individual peaks marked with asterisks. Periods of apnea are indicated by the black segments of the signal.

Each subject completed two standardized breath-holding protocols and across the full cohort, breath-holding events within the 10–20 s range were categorized by the operator as short-duration apnea-like events, while interruptions lasting 20–30 s were categorized as long-duration apnea-like events, depending on the onset and termination of the breath-holding. Figure 7 presents the final processed respiratory signals from subject 3 recorded in the supine, lateral, and prone positions during normal-breathing, short duration and long-duration breathing cessation events. In the supine position, normal breathing is characterized by regular, high-amplitude periodic oscillations, reflecting unobstructed respiratory motion. During short-duration apnea, a transient reduction in signal amplitude is observed, corresponding to the temporary cessation of breathing, followed by recovery to normal rhythmic patterns. Long-duration breathing cessation events produce a more pronounced and sustained suppression of the respiratory signal, clearly distinguishable from both normal-breathing and short-duration breathing-cessation events. In the lateral position, the respiratory signal during normal breathing exhibits slightly reduced amplitude and altered waveform morphology due to changes in chest wall motion; however, periodic breathing cycles remain clearly identifiable. Short-duration cessation in this posture is marked by a noticeable attenuation of the oscillatory pattern, while long-duration apnea results in an extended interval of near-complete signal suppression. Despite these position-dependent changes, the transition between normal-breathing and apnea-like events remain clearly resolved. In the prone position, respiratory motion amplitude is further reduced, reflecting restricted chest expansion. Nevertheless, normal breathing still produces discernible periodic oscillations in the extracted signal.

Across all subjects tested under resting conditions, measured breathing rates ranged from 12 to 18 BPM, consistent with established physiological norms for healthy adults. During normal breathing, mean respiratory rates were 19.08 ± 3.59 BPM in the supine position (*n* = 10), 17.59 ± 3.81 BPM in the lateral position (*n* = 6), and 16.56 ± 4.28 BPM in the prone position (*n* = 7). During short-term apnea-like events, respiratory rates decreased significantly to 12.90 ± 3.22 BPM in the supine position, 13.06 ± 2.10 BPM in the lateral position, and 13.54 ± 2.54 BPM in the prone position, representing a moderate reduction in breathing frequency corresponding to brief episodes of breathing cessation. During long-duration apnea-like events, respiratory rates showed the most substantial decrease, converging to approximately 10 BPM across all positions: 10.10 ± 1.45 BPM in the supine position, 10.12 ± 1.10 BPM in the lateral position, and 10.40 ± 1.85 BPM in the prone position. The hierarchical pattern of respiratory suppression with highest rates during normal breathing, intermediate rates during short-term cessations and lowest rates during long-duration cessations was observed across all three body positions, indicating robust and reliable detection of apnea-like events by the optical biosensing system. Additionally, body position significantly influenced baseline respiratory dynamics, with supine positioning consistently associated with the highest respiratory rates, lateral positioning with intermediate values, and prone positioning with the lowest rates. The lower standard deviations observed during long-duration cessations (1.10–1.85 BPM), compared to normal breathing (3.59–4.28 BPM), primarily reflect the physiological uniformity of sustained breath-holding. During prolonged cessations, chest wall motion is consistently suppressed across subjects, minimizing inter-subject variability despite differences in physical characteristics. In contrast, the higher variability during normal breathing arises from genuine individual differences in resting respiratory patterns, which are influenced by factors such as body composition, fitness level, and arousal state.

To quantify the extent to which each breathing cessation event was captured, sensitivity was computed in apnea-like events (23 short duration and 23 long-duration breathing cessations, total 46 events) and specificity was computed in 23 normal-breathing events, following the protocols mentioned in Section 2.4. To do so, a normalized threshold THR of the breathing signal is selected at which the system can reliably separate breathing cessation from normal respiratory activity, thereby enabling correct classification of each recording. To select the THR without introducing selection bias and to evaluate classification performance across all 14 participants, a nested cross-validation procedure was applied at the participant level. The 14 participants were partitioned into five groups of approximately equal size, constituting an 80/20 split in which 80% of the cohort formed the training set and the remaining 20% formed the held-out test set for each fold. This 80/20 partition was rotated across five folds so that every participant appeared in the test set exactly once, ensuring full cohort coverage without any participant influencing the THR selection for the fold in which they were tested. Within each outer fold, THR selection was performed using an inner cross-validation loop applied exclusively to the training participants: the six threshold values were scored on the inner training data and the threshold yielding the highest short-cessation sensitivity was selected, with long-cessation sensitivity as the tie-breaking criterion. The five folds were: Fold 1 (S01, S11, S13), Fold 2 (S04, S07, S08), Fold 3 (S02, S06, S09), Fold 4 (S05, S10, S12), and Fold 5 (S03, S14). The selected threshold was then applied to the held-out test participants to obtain unbiased performance estimates. In our experiments, a higher sensitivity was prioritized to minimize false negatives (missed detections) over false positives, ensuring reliable detection of all apnea-like events, while tolerating a moderate reduction in specificity. In our cohort, we obtained an optimized threshold of ${THR}_{opt}=0.235A_{max}$.

**Figure 8:**
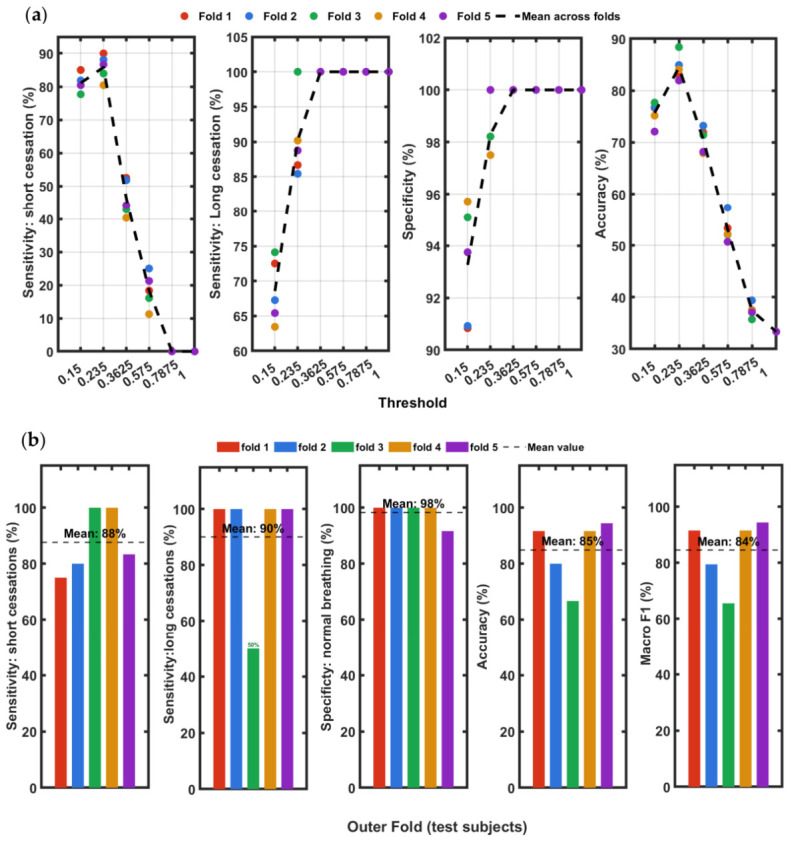
Test-set subject-wise classification performance. (**a**) Inner cross-validation performance curves for six threshold values across the five outer folds. The optimum threshold ${THR}_{opt}$ selected for each fold is 0.235 $\times \;A_{max}$ in all cases. At higher thresholds, some data points overlap. (**b**) Per fold test results from the participant-level five-fold cross-validation with the selected THR = 0.235. The black dashed line indicates the mean performance across all five folds.

Figure 8a shows the inner cross-validation performance curves for all six thresholds evaluated across the five outer folds. Short sensitivity peaks sharply at ${THR}_{opt}$ across all folds and drops steeply beyond this value, with near-zero sensitivity at thresholds of $0.575A_{max}$ and above. At the tightest threshold ($0.15A_{max}$), sensitivity ranges from 78 to 85% across folds, reflecting cases where signal suppression during breath-holding does not reach the threshold level. The normal-breathing specificity, which increases from approximately 91–95% at the tightest threshold to 100% from $0.3625A_{max}$ onwards, consistent across all folds. The overall accuracy peaks at ${THR}_{opt}$ ranging 81–89% across folds and declines monotonically at more permissive thresholds. The convergence of all fold lines at the ${THR}_{opt}=0.235A_{max}$ peak confirms this as the global optimum balancing sensitivity and specificity. Figure 8b shows the test-set classification performance for each outer fold across five metrics: overall accuracy, short-cessation sensitivity, long-cessation sensitivity, normal- breathing specificity, and Macro F1. Each colored bar represents one outer fold, identified on the x-axis by the held-out test participants, and the black dashed line indicates the mean across all five folds. Overall accuracy ranged from 66.7% (Fold 3: S02, S06, S09) to 94.4% (Fold 5: S03, S14), with a mean of 84.9±11.6% (95% CI:[78.2%,91.6%]). The lower accuracy in Fold 3 is attributable to S06, whose speckle displacement signal during the long breath-hold did not sustain a sub-threshold interval of 10 s or more at the selected threshold. Short-cessation sensitivity ranged from 75.0% (Fold 1) to 100% (Folds 3 and 4), with a mean of 87.7±11.6% (95% CI:[81.0%,94.4%]) reflecting the narrower detection window for 10–20 s cessations. Long-cessation sensitivity was 100% in four of five folds and 50% in Fold 3 due to the missed detection of S06, giving a mean of 90.0±22.4% (95% CI:[77.1%,100%]). Normal-breathing specificity was 100% in four folds and 91.7% in Fold 5, with a mean of 98.3±3.7% (95% CI:[96.2%, 100%]).

To verify the robustness of the threshold and assess whether classification performance is consistent across the full diversity of recorded events, a second nested cross- validation was conducted using a class-wise stratified splitting strategy. Rather than grouping by participant, the 69 recordings were distributed across five folds such that each fold received an approximately equal number of recordings from each session class: normal, short cessation, and long cessation. The 23 recordings of each class were independently shuffled and assigned to folds by interleaved indexing, yielding normal (5), short (5), and long (5) (15 recordings) in folds 1–3 and normal (4), short (4), and long (4) (12 recordings) in folds 4–5. This stratified construction ensures every fold presents a balanced classification problem, so fold-to-fold performance variation reflects genuine threshold sensitivity rather than accidental class imbalance. Threshold selection within each outer fold used the same nested inner cross-validation procedure as the participant-level approach.

**Figure 9:**
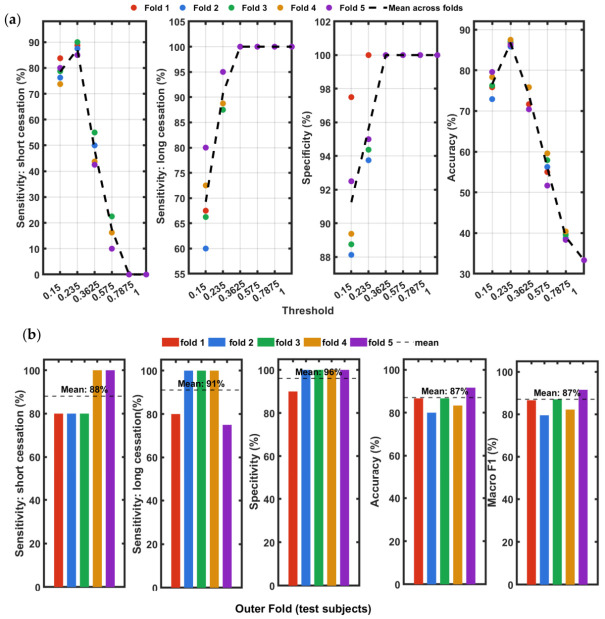
Test-set class-wise classification performance. (**a**) Inner cross-validation performance curves for all six threshold values across the five outer folds. The optimum threshold ${THR}_{opt}$ selected for each fold is 0.235 $\times \;A_{max}$ in all cases. At higher thresholds, some data points overlap. (**b**) Per outer-fold test results from the participant-level five-fold cross-validation with the selected ${THR}_{opt}$. The black dashed line indicates the mean performance across all five folds.

Figure 9a presents the inner cross-validation performance curves for the class-wise procedure. The fold lines show notably tighter agreement compared to Figure 8a, where the inner cross validation short-cessation sensitivity at ${THR}_{opt}=0.235\times A_{max}$ ranges from 85.0% to 90.0% across folds, compared to 80.4–90.0% in the participant-level procedure. The specificity curves converge to 100% from $0.3625\;A_{max}$ onwards, and accuracy peaks at ${THR}_{opt}$ in all folds (range 84.2–87.1%), confirming the same global optimum identified by both methods. Figure 9b presents the classification performance for each of the five stratified outer folds. In contrast to the participant-level results, the fold-to-fold variation is substantially lower, with overall accuracy ranging from 80.0% (Fold 2) to 91.7% (Fold 5), yielding a mean of 87.0±3.0% (95% CI: [85.3%, 88.7%]). This tighter spread reflects the balanced class composition of each fold: fold-to-fold variation is driven by threshold sensitivity rather than the participants tested. Short-cessation sensitivity ranged from 80.0% in Folds 1–3 to 100% in Folds 4–5, with a mean of 88.0±11.0% (95% CI:[81.6%, 94.4%]). Long-cessation sensitivity was 100% in four of five folds and 75% in Fold 5, giving a mean of 91.0±12.4% (95% CI:[83.8%, 98.2%]). Normal-breathing specificity was 100% in four folds and 90% in Fold 1, yielding a mean of 96.0±8.9% (95% CI:[90.9%, 100%]).

The Macro F1 score reported in Figure 8b and Figure 9b provides a balanced summary of classification performance across all three session categories (normal breathing, short and long cessation). For this three-class problem, the Macro F1 is defined as the unweighted mean of the per-class F1 scores (Equation (8)). A Macro F1 of 84.5% in subject-wise and 86.9% in class-wise cross-validation scheme indicates that the classifier achieves a consistent balance between precision and recall across the three categories at ${THR}_{opt}$. Similarly, the close agreement between the overall accuracy participant-level validation and class-level validation suggests that performance is uniformly distributed across classes and not dominated by any single category.

**Figure 10:**
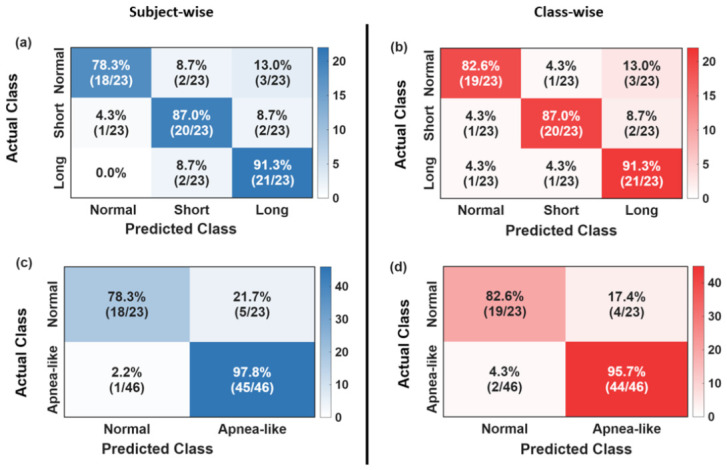
Aggregated confusion matrix across all participants from (**a**) participant-level and (**b**) class-wise using five-fold cross-validation at ${THR}_{opt}$. Binary confusion matrices derived by collapsing short and long cessation into a single apnea-like class for (**c**) subject-wise and (**d**) class-wise.

Figure 10 presents the confusion matrices from both cross-validation strategies in both three-class and binary forms. In all cases, each of the 69 recordings was tested exactly once across the five outer folds, and the predicted labels at the inner cross-validation selected threshold of 0.235 were accumulated into a single count matrix. In Figure 10a,b, each of the 69 recordings was tested exactly once across the five outer folds, and the predicted labels at the inner cross validation selected threshold were accumulated into a single 3 × 3 count matrix. In the subject-wise evaluation (Figure 10a), for normal breathing, 18 out of 23 recordings were correctly identified and 5 were misclassified as cessation events (2 as short and 3 as long). For short cessation, 20 out of 23 recordings were correctly classified (TP), 1 was completely missed (FN, predicted as normal), and 2 were assigned to the wrong duration category (predicted as long). Crucially, these 2 misclassifications represent successful detection of a cessation event but assigned the event to the wrong duration criteria. For long cessation, all 23 recordings were detected as containing a cessation event (zero FN), with 21 correctly classified as long (TP) and 2 labeled as short (misclassification), yielding 91.3% sensitivity. Overall, 59 out of 69 recordings were correctly classified, giving an accuracy of 85.5%. The class-wise evaluation (Figure 10b) produces similar results with 60 out of 69 recordings correctly classified, giving an accuracy of 86.9%, confirming that the aggregated outcome is independent of the fold-splitting strategy.

Figure 10c,d present the binary confusion matrices derived by collapsing the three-class predictions into two categories: normal and apnea-like, where the latter combines both short- and long-cessation events. This collapsing is clinically motivated from a diagnostic standpoint, where the primary objective of the system is to reliably flag the presence of any apnea-like breathing cessations, with duration subtyping being a secondary refinement. Under this binary view, the duration misclassifications that penalized the three-class sensitivity are correctly absorbed into the true positive count, since the event was still flagged as apnea-like regardless of which subtype was predicted.

It is important to distinguish between two related but conceptually distinct quantities that both appear in the confusion matrix analysis: normal-class recall and normal-breathing specificity. Normal-class recall (also called sensitivity of the normal class) is observed directly from the confusion matrix as the fraction of true normal recordings that were correctly predicted as normal (18/23 = 78.3% for subject-wise and 19/23 = 82.6% for class-wise) and measures how well the system recognizes normal breathing when it occurs. Apnea-like detection sensitivity is the fraction of true apnea-like recordings correctly flagged as apnea-like, which is 45/46 = 97.8% (subject-wise, Figure 10c) and 44/46 = 95.7% (class-wise, Figure 10d) and measures how rarely the system fails to detect a genuine cessation event. On the other hand, specificity (as reported in Figure 8 and Figure 9) is a system-level metric computed fold-by-fold across all non-target classes and averaged across the five outer folds, and therefore cannot be read directly from a single row or column of the aggregated confusion matrix. The small numerical difference between the fold-averaged specificity (subject-wise) of 98.3 ± 3.7% and the binary apnea detection sensitivity of 97.8% arises because the five outer folds contain unequal numbers of recordings, so each fold contributes unequally to the flat-count result but equally to the fold average.

**Figure 11:**
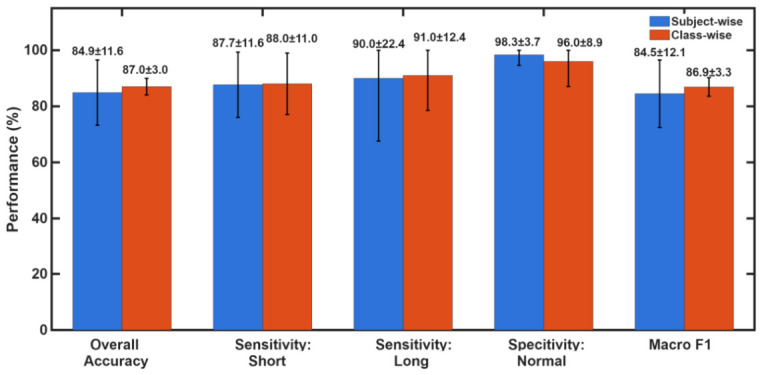
Direct comparison of mean classification performance between subject-wise (blue) and class-wise stratified (red) nested five-fold cross-validation at the selected ${THR}_{opt}$.

Figure 11 provides a comparison of the mean classification performance between the two nested cross-validation strategies across all five metrics. Across all five metrics, the two strategies produce closely aligned mean values. Overall accuracy was 84.9 ± 11.6% (95% CI:[78.2%,91.6%]) for subject-wise and 87.0±3.0% (95% CI: [85.3%, 88.7%]) for class-wise evaluation. Macro F1 was 84.5 ± 12.1% and 86.9 ± 3.3%. The convergence of mean values across two structurally different validation procedures confirms that the reported performance figures are a stable property of the detection algorithm and not an artefact of any fold-assignment strategy. The most informative feature of Figure 11 is the contrast in standard deviation (SD) between the two methods. The subject-wise procedure consistently yields larger standard deviations across all metrics: accuracy SD of 11.6% versus 3.0% for class-wise, long-cessation sensitivity SD of 22.4% versus 12.4%, and Macro F1 SD of 12.1% versus 3.3%. This is so because in subject-wise splitting, entire participants are held out together, meaning that one of the folds may by chance contain participants whose physiological signals are atypical, for example, as occurred in fold 3 (S02, S06, S09), where S06’s signal behavior drove accuracy down to 66.7% and long-cessation sensitivity to 50%, inflating the SD for both metrics. In class-wise classification, each fold receives a stratified sample of recordings drawn from all participants, so no fold is dominated by the characteristics of any single individual. The result is that fold-to-fold variation in class-wise cross validation reflects genuine threshold sensitivity across the diversity of recorded events, whereas fold-to-fold variation in subject-wise cross validation does not allow inter-subject heterogeneity. Notably, normal-breathing specificity shows an opposite SD pattern: 3.7% for subject-wise versus 8.9% for class-wise. In subject-wise cross validation, the four folds containing three subjects each produced 100% specificity, with only fold 5 (two subjects) yielding 91.7%, giving a tight spread. In class-wise cross validation, fold 1 produced 80% specificity, while all other folds reached 100%, resulting in a wider fold-to-fold spread despite the overall mean remaining high. This observation underscores that even under balanced stratification, random variation in which specific recordings fall into a given fold can introduce metric variability, particularly for metrics sensitive to a small number of borderline cases. Taken together, the two SD profiles in Figure 11 provide complementary diagnostic information: the subject-wise SD characterizes robustness to new unseen participants, while the class-wise SD characterizes robustness to the specific mix of recording events presented at test time.

## 4. Discussion

This study demonstrated the feasibility of non-contact secondary laser speckle pattern analysis for detecting apnea-like breathing cessations across a cohort of 14 healthy participants tested in three sleeping positions. At the recommended optimized detection threshold of ${THR}_{opt}=0.235\;\times \;A_{max}$, our system consistently separates breathing cessations (long/short) from normal respiratory activity, as confirmed by two independent nested cross-validation procedures. The participant-level procedure yielded an overall accuracy of 84.9 ± 11.6% (95% CI: [78.2%, 91.6%]), and the class-wise stratified procedure yielded 87.0 ± 3.0% (95% CI: [85.3%, 88.7%]). The near-identical means and the convergent threshold selection across 80%/20% independent training sets confirm that the reported performance is a stable property of the detection system. The long-cessation detection rate is the strongest result from this study in comparison to short-cessations, which is expected given the narrower 10–20 s suppression window. However, this result is clinically meaningful because prolonged apnea events are associated with the most severe oxygen desaturation and therefore the highest priority for detection in a screening context. Only 1 of 23 short cessation recordings were missed entirely; the 2 misclassification cases (short detected as long or vice versa) represent successful detections assigned to the wrong duration category and do not constitute missed apnea events. Collapsing to a binary framework, where short and long breathing cessations are categorized as apnea-like event, demonstrates that the system achieves high apnea-like event detection sensitivity (97.8% in subject-wise and 95.7% class-wise), confirms the reliability for primary apnea screening. The wide confidence interval for short-cessation sensitivity reflects the modest sample size of 23 recordings per class and highlights the need for larger clinical validation studies. Five of 23 normal recordings triggered a false positive detection, arising from participants whose resting breathing trough amplitude occasionally fell below the ${THR}_{opt}$ for 10 s or more. This inter-subject variability in resting chest wall motion amplitude is the primary source of false positives and represents the main limitation of the fixed-fraction threshold approach. Future work should investigate threshold calibration in which the threshold fraction is personalized based on the individual’s resting breathing amplitude distribution.

The consistent threshold selection across all ten outer folds (five subject-wise, five class-wise) confirms that ${THR}_{opt}$ represents the optimal operating point for this speckle-based respiratory monitoring system, regardless of the specific composition of the training data. This stability suggests that the threshold reflects a genuine physical property of the speckle signal during breath-holding rather than a value overfitted to a particular sample of participants.

Several limitations of the current study should be acknowledged. First, the experiments were conducted on healthy volunteers performing voluntary breath-holds rather than patients with clinically diagnosed sleep apnea. As a result, the mechanical signature of voluntary cessation may differ from that observed in real pathological conditions which can be clinically diagnosed only with PSG. In obstructive sleep apnea (OSA), breathing cessation is typically associated with gradual airway collapse and continued respiratory effort, often resulting in irregular or paradoxical chest movements. In contrast, central sleep apnea (CSA) is characterized by a complete absence of respiratory effort and chest wall motion. The current system, which detects complete cessation of chest wall displacement, is therefore more representative of CSA-like events. This presents an opportunity for future development. By integrating machine learning techniques, the system could potentially identify subtle deviations in breathing patterns, enabling detection of OSA-related similarities in breathing abnormalities in addition to CSA-like events. Such an approach would significantly enhance the diagnostic capability of the system. While our system can detect apnea-like breathing cessations, this system is not a clinical replacement for sleep apnea that requires clinical diagnosis by now.

Secondly, the cohort size of 14 participants, while sufficient for demonstrating feasibility, is limited for clinical validation. This is reflected in the relatively wide confidence intervals observed for short-cessation sensitivity and normal-breathing specificity. In addition, the study lacked demographic diversity, with only one female participant included, and a relatively narrow BMI range (19.6–28.7 kg/m^2^), limiting the generalizability of the findings. The study also did not include individuals with clinically diagnosed sleep apnea, morbid obesity, or other relevant comorbidities, which is a next step in our research.

Third, all experiments were performed under controlled laboratory conditions with dim ambient illumination. The participants were not in natural sleep but instead simulated typical sleep postures. In real-world settings, variations in ambient lighting, surface reflectance, and involuntary body movements during sleep are likely to affect signal quality and system performance. These factors were not captured in the present study and warrant further investigation. Another key limitation of the present study is that although the BIOPAC reference signal was continuously monitored and the onset and termination of artificially induced apnea events were verified by three independent observers, a comprehensive quantitative comparison between our optical system and the BIOPAC reference across all subjects was not performed. This was primarily due to the use of an analog BIOPAC recording setup in most sessions, which limited the availability of digital data required for systematic statistical. Future work will address this limitation by incorporating fully digitized, time-synchronized reference recordings, enabling rigorous quantitative validation of onset and offset detection performance across all participants. This will allow the use of standardized agreement analyses, including correlation metrics to more comprehensively assess the agreement between the proposed optical system and the reference standard.

From an algorithmic perspective, certain normal-breathing recordings were incorrectly classified as apnea-like events. This primarily occurred due to variability in breathing amplitude within a recording. Specifically, isolated high-amplitude peaks can increase $A_{\max}$, causing subsequent normal-breathing cycles to fall below the selected threshold of ${THR}_{opt}$ even in the absence of true cessation. This highlights a limitation of global maximum-based normalization, which does not fully account for intra-recording variability. Future improvements should consider adaptive or locally normalized thresholding approaches to enhance robustness. Furthermore, the current implementation evaluates recordings over fixed 60 s intervals and is designed to detect the presence of a single apnea-like event within that window. While this approach is suitable for controlled validation, it does not reflect real sleep conditions, where multiple cessation events may occur over extended periods. Future work should therefore focus on continuous monitoring over longer durations to enable detection of multiple events and their temporal distribution.

Another important consideration is the classification framework adopted in this study. Unlike clinical practice, which relies on the Apnea–Hypopnea Index (AHI) based on event frequency, this work uses a duration-based classification of individual cessation events. While event duration is clinically meaningful since longer cessation periods are associated with greater oxygen desaturation and increased physiological risk, the primary contribution of this study is demonstrating reliable detection of individual apnea-like events. This capability is a fundamental prerequisite for any frequency-based classification. Future work should therefore extend the system to continuous overnight monitoring, allowing computation of AHI (events per hour) and direct comparison with polysomnography (PSG)-derived metrics.

Finally, the current algorithm implementation performs offline processing. Future work should consider machine learning and algorithm enhancement for continuous evaluation of speckle data. The main bottleneck is the real-time correlation. It can be done via optimized Fourier-based correlation (using FTT programming packet). The rough calculation of this complexity is O(*N* × *M*log(*N* × *M*)). Therefore, by having *N* × *M* = 265 × 126 pixels of ROI, we can yield ~150 K operations/s to perform this correlation, which is fully feasible by modern computers.

Despite these limitations, the proposed system offers several advantages over existing technologies. While polysomnography (PSG) remains the gold-standard diagnostic tool for sleep apnea, it requires overnight laboratory attendance, multiple body-attached sensors, and trained personnel, limiting its accessibility and making it unsuitable for large-scale screening. It is estimated that approximately 80% of individuals with clinically significant sleep apnea remain undiagnosed. Commercial contactless systems, such as under-mattress sensors (e.g., EarlySense), have addressed some of these accessibility challenges by enabling passive home monitoring. These systems rely on piezoelectric or pneumatic sensing to detect respiratory micro-vibrations transmitted through the mattress. However, such signals are spatially averaged across the contact surface, making it difficult to distinguish between multiple occupants sharing a bed. Additionally, these systems cannot reliably detect discrete chest wall cessation events or classify them based on duration. In contrast, the proposed laser speckle system directly measures localized chest wall displacement by targeting a specific region on an individual’s body. This enables subject-specific monitoring and allows independent assessment of multiple individuals using separate optical units. The system operates in a fully contactless manner, is independent of mattress properties, and remains effective across different sleeping positions. Importantly, it enables detection of complete cessation events and classification into short-duration (10–20 s) and long-duration (20–30 s) categories. From a clinical perspective, the system is best positioned as an early-stage screening tool rather than a standalone diagnostic method. By identifying repeated cessation patterns, it can serve as a warning system and prompt timely referral for formal PSG-based diagnosis, thereby bridging the gap between no monitoring and full clinical evaluation.

Another promising extension of this technology is pediatric sleep monitoring. Children and infants exhibit higher baseline respiratory rates compared to adults—typically 20–30 breaths per minute in children and 30–60 breaths per minute in infants. By adapting the algorithm to account for these age-dependent physiological differences, the system could be tailored for non-contact respiratory monitoring in pediatric populations, where conventional methods are often more challenging to implement.

The system’s non-contact, non-invasive design, together with its low-cost hardware implementation and simple signal processing pipeline, makes it a promising candidate for home-based sleep apnea screening. The next critical step toward clinical translation is validation against polysomnography in clinically diagnosed patient cohorts.

## 5. Conclusions

This study demonstrated that far-field laser speckle pattern analysis can reliably detect apnea-like breathing cessations in healthy volunteers under controlled laboratory conditions. A normalized detection threshold of 0.235 $\times \;A_{max}$ was identified as the optimal operating point through a rigorously bias-free nested cross-validation procedure. Across all 69 recordings covering all 14 participants, the system achieved overall accuracy of 87.0 ± 3.0% (95% CI: [85.3%, 88.7%]) using class-wise cross fold verification and 84.9 ± 11.6% (95% CI: [78.2%, 91.6%]) using subject-wise stratification. The Macro F1 score of 84.5 ± 12.1% (subject-wise) and 86.9 ± 3.3% (class-wise) confirms balanced classification performance across all three session categories. The model consistently detects apnea-like events with very high sensitivity (97.8% subject-wise and 95.7% class-wise), supporting its effectiveness as a screening tool. These results demonstrate that the proposed system reliably detects the mechanical signature of breathing cessation across diverse participant body characteristics and three sleeping positions (supine, lateral, prone) without any physical contact.

## Data Availability

The datasets presented in this article are not readily available because privacy reasons.

## References

[1] Leger D., Bayon V., Laaban J.P., Philip P. (2012). Impact of Sleep Apnea on Economics. Sleep Med. Rev..

[2] Newman A.B., Nieto F.J., Guidry U., Lind B.K., Redline S., Shahar E., Pickering T.G., Quan S.F. (2001). Relation of Sleep-Disordered Breathing to Cardiovascular Disease Risk Factors: The Sleep Heart Health Study. Arch. Intern. Med..

[3] Stansbury R.C., Strollo P.J. (2015). Clinical Manifestations of Sleep Apnea. J. Thorac. Dis..

[4] Kim K.B., Movahed R., Malhotra R.K., Stanley J.J. (2017). An Evidence-Based, Multidisciplinary Textbook Management of Obstructive Sleep Apnea.

[5] Sönmez I., Vo Dupuy A., Yu K.S., Cronin J., Yee J., Azarbarzin A. (2025). Unmasking Obstructive Sleep Apnea: Estimated Prevalence and Impact in the United States. Respir. Med..

[6] Benjafield A.V., Ayas N.T., Eastwood P.R., Heinzer R., Ip M.S.M., Morrell M.J., Nunez C.M., Patel S.R., Penzel T., Pépin J.-L. (2019). Estimation of the Global Prevalence and Burden of Obstructive Sleep Apnea: A Literature-Based Analysis. Lancet Respir. Med..

[7] Gottlieb D.J., Punjabi N.M. (2020). Diagnosis and Management of Obstructive Sleep Apnea: A Review. J. Am. Med. Assoc..

[8] Lévy P., Kohler M., McNicholas W.T., Barbé F., McEvoy R.D., Somers V.K., Lavie L., Pépin J.L. (2015). Obstructive Sleep Apnea Syndrome. Nat. Rev. Dis. Primers.

[9] Senaratna C.V., Perret J.L., Lodge C.J., Lowe A.J., Campbell B.E., Matheson M.C., Hamilton G.S., Dharmage S.C. (2017). Prevalence of Obstructive Sleep Apnea in the General Population: A Systematic Review. Sleep Med. Rev..

[10] Donovan L.M., Kapur V.K. (2016). Prevalence and Characteristics of Central Compared to Obstructive Sleep Apnea: Analyses from the Sleep Heart Health Study Cohort. Sleep.

[11] White D.P. (2005). Pathogenesis of Obstructive and Central Sleep Apnea. Am. J. Respir. Crit. Care Med..

[12] Badr M.S., Javaheri S. (2019). Central Sleep Apnea: A Brief Review. Curr. Pulmonol. Rep..

[13] Eckert D.J., Jordan A.S., Merchia P., Malhotra A. (2007). Central Sleep Apnea: Pathophysiology and Treatment. Chest.

[14] Randerath W., Verbraecken J., Andreas S., Arzt M., Bloch K.E., Brack T., Buyse B., De Backer W., Eckert D.J., Grote L. (2017). Definition, Discrimination, Diagnosis and Treatment of Central Breathing Disturbances During Sleep. Eur. Respir. J..

[15] Dernaika T., Tawk M., Nazir S., Younis W., Kinasewitz G.T. (2007). The Significance and Outcome of Continuous Positive Airway Pressure-Related Central Sleep Apnea During Split-Night Sleep Studies. Chest.

[16] Sateia M.J. (2014). International Classification of Sleep Disorders—Third Edition: Highlights and Modifications. Chest.

[17] Berry R.B., Budhiraja R., Gottlieb D.J., Gozal D., Iber C., Kapur V.K., Marcus C.L., Mehra R., Parthasarathy S., Quan S.F. (2012). Rules for Scoring Respiratory Events in Sleep: Update of the 2007 AASM Manual for the Scoring of Sleep and Associated Events. J. Clin. Sleep Med..

[18] Oksenberg A., Leppänen T. (2023). Duration of Respiratory Events in Obstructive Sleep Apnea: In Search of Paradoxical Results. Sleep Med. Rev..

[19] Butler M.P., Emch J.T., Rueschman M., Sands S.A., Shea S.A., Wellman A., Redline S. (2019). Apnea-Hypopnea Event Duration Predicts Mortality in Men and Women in the Sleep Heart Health Study. Am. J. Respir. Crit. Care Med..

[20] Collop N.A., Anderson W.M., Boehlecke B., Claman D., Goldberg R., Gottlieb D.J., Hudgel D., Sateia M., Schwab R., Portable Monitoring Task Force of the American Academy of Sleep Medicine (2007). Clinical Guidelines for the Use of Unattended Portable Monitors in the Diagnosis of Obstructive Sleep Apnea in Adult Patients. J. Clin. Sleep Med..

[21] Kapur V.K., Auckley D.H., Chowdhuri S., Kuhlmann D.C., Mehra R., Ramar K., Harrod C.G. (2017). Clinical Practice Guideline for Diagnostic Testing for Adult Obstructive Sleep Apnea: An American Academy of Sleep Medicine Clinical Practice Guideline. J. Clin. Sleep Med..

[22] Boulos M.I., Jairam T., Kendzerska T. (2019). Normal Polysomnography Parameters in Healthy Adults: A Systematic Review and Meta-Analysis. Lancet Respir. Med..

[23] Goldstein C., Ghanbari H., Sharma S., Collop N., Namen A., Kirsch D.B., Drucker M., Khayat R., Pollock M., Torstrick B. (2025). Polysomnography Validation of SANSA to Detect Obstructive Sleep Apnea. Front. Neurol..

[24] Flemons W.W., Douglas N.J., Kuna S.T., Rodenstein D.O., Wheatley J. (2004). Access to Diagnosis and Treatment of Patients with Suspected Sleep Apnea. Am. J. Respir. Crit. Care Med..

[25] Corral J., Sanchez-Quiroga M.A., Carmona-Bernal C., Sanchez-Armengol A., De La Torre A.S., Duran-Cantolla J., Egea C.J., Salord N., Monasterio C., Teran J. (2017). Conventional Polysomnography Is Not Necessary for the Management of Most Patients with Suspected Obstructive Sleep Apnea: Noninferiority, Randomized Controlled Trial. Am. J. Respir. Crit. Care Med..

[26] Osa-Sanchez A., Ramos-Martinez-de-Soria J., Mendez-Zorrilla A., Ruiz I.O., Garcia-Zapirain B. (2025). Wearable Sensors and Artificial Intelligence for Sleep Apnea Detection: A Systematic Review. J. Med. Syst..

[27] Abd-Alrazaq A., Aslam H., AlSaad R., Alsahli M., Ahmed A., Damseh R., Aziz S., Sheikh J. (2024). Detection of Sleep Apnea Using Wearable AI: Systematic Review and Meta-Analysis. J. Med. Internet Res..

[28] Tran N.T., Tran H.N., Mai A.T. (2023). A Wearable Device for At-Home Obstructive Sleep Apnea Assessment: State-of-the-Art and Research Challenges. Front. Neurol..

[29] Weaver T.E., Chasens E.R. (2007). Continuous Positive Airway Pressure Treatment for Sleep Apnea in Older Adults. Sleep Med. Rev..

[30] Schwab R.J., Badr S.M., Epstein L.J., Gay P.C., Gozal D., Kohler M., Lévy P., Malhotra A., Phillips B.A., Rosen I.M. (2013). An Official American Thoracic Society Statement: Continuous Positive Airway Pressure Adherence Tracking Systems, the Optimal Monitoring Strategies and Outcome Measures in Adults. Am. J. Respir. Crit. Care Med..

[31] Karagiannis A., Tzitiridou M., Kafkia T., Kourakos M. (2023). Monitoring Seasonal Compliance of Patients with Obstructive Sleep Apnea Using CPAP Systems via SD Card. Acta Inform. Medica.

[32] Penzel T., Schöbel C., Fietze I. (2018). New Technology to Assess Sleep Apnea: Wearables, Smartphones, and Accessories. F1000Research.

[33] Charlton P.H., Kyriacou P.A., Mant J., Marozas V., Chowienczyk P., Alastruey J. (2022). Wearable Photoplethysmography for Cardiovascular Monitoring. Proc. IEEE.

[34] Ghamari M. (2018). A Review on Wearable Photoplethysmography Sensors and Their Potential Future Applications in Health Care. Int. J. Biosens. Bioelectron..

[35] Tal A., Shinar Z., Shaki D., Codish S., Goldbart A. (2017). Validation of Contact-Free Sleep Monitoring Device with Comparison to Polysomnography. J. Clin. Sleep Med..

[36] Ben-Ari J., Zimlichman E., Adi N., Sorkine P. (2010). Contactless Respiratory and Heart Rate Monitoring: Validation of an Innovative Tool. J. Med. Eng. Technol..

[37] Yang C., Wang Z., Xiao K., Ushakov N., Kumar S., Li X., Min R. (2024). Portable Optical Fiber Biosensors Integrated with Smartphone: Technologies, Applications, and Challenges. Biomed. Opt. Express.

[38] Mortazavi S., Makouei S., Abbasian K., Danishvar S. (2025). Emerging Trends in Optical Fiber Biosensing for Non-Invasive Biomedical Analysis. Photonics.

[39] Vavrinsky E., Esfahani N.E., Hausner M., Kuzma A., Rezo V., Donoval M., Kosnacova H. (2022). The Current State of Optical Sensors in Medical Wearables. Biosensors.

[40] Que S., van Meulen F., Verkruijsse W., van Gastel M., Overeem S., Zinger S., Stuijk S. (2023). Speckle Vibrometry for Instantaneous Heart Rate Monitoring. Sensors.

[41] Palomares D.E., Tran P.L., Jerman C., Momayez M., Deymier P., Sheriff J., Bluestein D., Parthasarathy S., Slepian M.J. (2023). Vibro-Acoustic Platelet Activation: An Additive Mechanism of Prothrombosis with Applicability to Snoring and Obstructive Sleep Apnea. Bioengineering.

[42] Que S., Cramer I., Dekker L., Overeem S., Bouwman A., Zinger S., Stuijk S., van Meulen F. (2024). Speckle Vibrometry for Contactless Instantaneous Heart Rate and Respiration Rate Monitoring on Mechanically Ventilated Patients. Sensors.

[43] Rothberg S.J., Allen M.S., Castellini P., Di Maio D., Dirckx J.J.J., Ewins D.J. (2017). An International Review of Laser Doppler Vibrometry: Making Light Work of Vibration Measurement. Opt. Lasers Eng..

[44] Povsic K., Mozina J., Jezersek M., Flezar M. (2012). Laser 3-D Measuring System and Real-Time Visual Feedback for Teaching and Correcting Breathing. J. Biomed. Opt..

[45] Wijenayake U., Park S.Y. (2017). Real-Time External Respiratory Motion Measuring Technique Using an RGB-D Camera and Principal Component Analysis. Sensors.

[46] Chen H., Cheng Y., Liu D., Zhang X., Zhang J., Que C., Wang G., Fang J. (2010). Color Structured Light System of Chest Wall Motion Measurement for Respiratory Volume Evaluation. J. Biomed. Opt..

[47] Dainty J.C. (1975). Laser Speckle and Related Phenomena (Topics in Applied Physics).

[48] Zaletelj K., Agrež V., Slavič J., Petkovšek R., Boltežar M. (2021). Laser-Light Speckle Formation for Deflection-Shape Identification Using Digital Image Correlation. Mech. Syst. Signal Process..

[49] Briers D., Duncan D.D., Hirst E., Kirkpatrick S.J., Larsson M., Steenbergen W., Stromberg T., Thompson O.B. (2013). Laser Speckle Contrast Imaging: Theoretical and Practical Limitations. J. Biomed. Opt..

[50] Boas D.A., Dunn A.K. (2010). Laser Speckle Contrast Imaging in Biomedical Optics. J. Biomed. Opt..

[51] Draijer M., Hondebrink E., Van Leeuwen T., Steenbergen W. (2009). Review of Laser Speckle Contrast Techniques for Visualizing Tissue Perfusion. Lasers Med. Sci..

[52] Zalevsky Z., Beiderman Y., Margalit I., Gingold S., Teicher M., Mico V., Garcia J. (2009). Simultaneous Remote Extraction of Multiple Speech Sources and Heart Beat from Secondary Speckles Pattern. Opt. Express.

[53] Beiderman Y., Blumenberg R., Rabani N., Teicher M., Garcia J., Mico V., Zalevsky Z. (2011). Demonstration of Remote Optical Measurement Configuration That Correlates to Glucose Concentration in Blood. Biomed. Opt. Express.

[54] Beiderman Y., Horovitz I., Burshtein N., Teicher M., Garcia J., Mico V., Zalevsky Z. (2010). Remote Estimation of Blood Pulse Pressure via Temporal Tracking of Reflected Secondary Speckles Pattern. J. Biomed. Opt..

[55] Kalyuzhner Z., Agdarov S., Beiderman Y., Zalevsky Z. (2025). Visual Cortex Speckle Imaging for Shape Recognition. Sci. Rep..

[56] Segal N., Kalyuzhner Z., Agdarov S., Beiderman Y., Zalevsky Z. (2025). AI-Powered Remote Monitoring of Brain Responses to Clear and Incomprehensible Speech via Speckle Pattern Analysis. J. Biomed. Opt..

[57] Goodman J.W. (2017). An Introduction to Fourier Optics.

[58] Goodman J.W. (2020). Speckle Phenomena in Optics: Theory and Applications.

[59] Massaroni C., Nicolò A., Lo Presti D., Sacchetti M., Silvestri S., Schena E. (2019). Contact-Based Methods for Measuring Respiratory Rate. Sensors.

[60] Rehouma H., Noumeir R., Essouri S., Jouvet P. (2020). Advancements in Methods and Camera-Based Sensors for the Quantification of Respiration. Sensors.

[61] Liang W.M., Ji Y.X., Xiao J., Truskauskaitė I., Hendrixson A., Bai Z.M., Ruksenas O. (2024). Respiratory Patterns and Physical Fitness in Healthy Adults: A Cross-Sectional Study. BMC Public Health.

[62] (2014). Safety of Laser Products—Part 1: Equipment Classification and Requirements, Ed 3.0.

